# TAK-272 (imarikiren), a novel renin inhibitor, improves cardiac remodeling and mortality in a murine heart failure model

**DOI:** 10.1371/journal.pone.0202176

**Published:** 2018-08-09

**Authors:** Tomoya Hara, Satoshi Nishimura, Toshihiro Yamamoto, Yumiko Kajimoto, Keiji Kusumoto, Ray Kanagawa, Shota Ikeda, Tomoyuki Nishimoto

**Affiliations:** Pharmaceutical Research Division, Takeda Pharmaceutical Company Limited, Fujisawa, Kanagawa, Japan; Max Delbruck Centrum fur Molekulare Medizin Berlin Buch, GERMANY

## Abstract

The renin-angiotensin system (RAS), which plays an important role in the progression of heart failure, is efficiently blocked by the inhibition of renin, the rate-limiting enzyme in the RAS cascade. In the present study, we investigated the cardioprotective effects of TAK-272 (SCO-272, imarikiren), a novel, orally effective direct renin inhibitor (DRI), and compared its efficacy with that of aliskiren, a DRI that is already available in the market. TAK-272 was administered to calsequestrin transgenic (CSQ-tg) heart failure mouse model that show severe symptoms and high mortality. The CSQ-tg mice treated with 300 mg/kg, the highest dose tested, of TAK-272 showed significantly reduced plasma renin activity (PRA), cardiac hypertrophy, and lung congestion. Further, TAK-272 reduced cardiomyocyte injury accompanied by an attenuation of the increase in NADPH oxidase 4 and nitric oxide synthase 3 expressions. TAK-272 also prolonged the survival of CSQ-tg mice in a dose-dependent manner (30 mg/kg: *P* = 0.42, 100 mg/kg: *P* = 0.12, 300 mg/kg: *P* < 0.01). Additionally, when compared at the same dose level (300 mg/kg), TAK-272 showed strong and sustained PRA inhibition and reduced the heart weight and plasma N-terminal pro-brain natriuretic peptide (NT-proBNP) concentration, a heart failure biomarker, while aliskiren showed a significant weaker PRA inhibition and failed to demonstrate any cardioprotective effects. Our results showed that TAK-272 is an orally active and persistent renin inhibitor, which reduced the mortality of CSQ-tg mice and conferred protection against cardiac hypertrophy and injury. Thus, TAK-272 treatment could provide a new therapeutic approach for heart failure.

## Introduction

Heart failure, which is characterized by cardiac dysfunction, remodeling, and death [1], is one of the most important causes of death globally [2, 3]. The renin-angiotensin system (RAS) plays an essential role in the pathophysiological progression of heart failure, which involves cardiac hypertrophy, apoptosis, and ventricular dilation [4, 5]. As the RAS is known to be activated in heart failure patients [6, 7], various therapeutic approaches involving RAS inhibition, including the use of angiotensin converting enzyme (ACE) inhibitors and angiotensin II receptor blockers (ARBs), have been developed in the past decades. Although these drugs have been effective in reducing the mortality and morbidity in heart failure patients in randomized controlled trials [8–11], they may not inhibit the RAS efficiently, as compensation and escape mechanisms may affect their efficacy [12]. The inhibition of renin, which controls the first and rate-limiting step in the RAS pathway that catalyzes the generation of angiotensin I from angiotensinogen, should efficiently block the RAS cascade even in cases of elevated plasma or tissue renin activities [13, 14]. Aliskiren, the first direct renin inhibitor (DRI) launched in 2007, is still the only DRI in the market despite low oral bioavailability (BA: 2.6%) in human beings [15]. Therefore, in order to develop DRIs with favorable pharmacokinetic (PK) profiles, we synthesized a novel, potent, and orally efficacious renin inhibitor, TAK-272 (SCO-272, imarikiren) [16]. The cardioprotective effects of aliskiren have been investigated in several animal models of myocardial infarction, pressure-overload, or doxorubicin-induced cardiac failure [17–19]. In the present study, we investigated the cardioprotective effects of TAK-272 in calsequestrin (CSQ) transgenic (tg) (CSQ-tg) heart failure mouse model. The overexpression of CSQ, a key protein for cardiac Ca^2+^ homeostasis, alters the intracellular Ca^2+^ cycling in cardiomyocytes, resulting in cardiac dysfunction [20–22]. CSQ-tg mice, which therefore show abnormal Ca^2+^ handling and various detrimental heart failure-like phenotypes including cardiac hypertrophy, fibrosis, and pump failure, are useful models for heart failure research [23–25]. Thus, we evaluated the effects of TAK-272 on cardiac remodeling and mortality in CSQ-tg mice, and compared the efficacy of the two renin inhibitors, TAK-272 and aliskiren.

## Materials and methods

### Drugs

TAK-272 was synthesized in Takeda Pharmaceutical (Kanagawa, Japan) and aliskiren was purchased from KNC Laboratories (Kobe, Japan). These compounds were dissolved in 0.5% (w/v) methylcellulose solution and administered by oral gavage at a dose of 10 ml/kg, once daily in all studies.

### Animals

The transgenic mice with cardiac-specific overexpression of canine CSQ were originally developed in a facility in the Indiana University School of Medicine [24] and then bred in Takeda Rabics (Osaka, Japan). The transgenic line was created on a hybrid (C57BL/6J × DBA/2N) F1 background. Female CSQ-tg mice were randomized based on their body weights in all experiments. All animal experiments were approved by the Institutional Animal Care and Use Committee of Shonan Research Center, Takeda Pharmaceutical Company Limited.

### Survival study

The CSQ-tg mice were treated with either 0.5% (w/v) methylcellulose solution (vehicle) (n = 20) or TAK-272 (30, 100, and 300 mg/kg, n = 20 in each dose group). Body weight was measured twice a week and mice condition was monitored once a day. After 7 weeks of age, their rectal temperatures were also monitored once daily until the end of study (36 days after starting treatment). All research staffs were trained to enable to measure rectal temperature with consistent technique. As a humane endpoint, the animals were euthanized immediately by carbon dioxide when their rectal temperatures dropped below 29°C to minimize their suffering and distress, according to pre-specified criteria. Mice with clinical symptoms such as extremely reduced physical activity and body weight loss were also euthanized. Severe hypertrophy was observed in all carcasses as necropsy findings, while no obvious changes of other organs such as liver, kidney, and brain were found by the visual check. From these findings, we consider that these mice were dead by a heart failure as a primal cause. Forty-four mice were euthanized and seventeen mice were found dead in eighty mice which we used in this study.

### Cardioprotective effects of TAK-272

The CSQ-tg mice were treated with either vehicle (n = 10) or TAK-272 (30, 100, and 300 mg/kg, n = 10 in each dose group) from 5 weeks of age for evaluating the cardioprotective effects of TAK-272. After 19 days of drug administration, blood samples were collected from the abdominal vein using EDTA (15575020, Thermo Fisher Scientific K.K., Japan) (at a final concentration of 3 mM) as an anticoagulant under anesthesia with 2–3% isoflurane (Mylan, UK). After sacrificing the animals with bleeding, the hearts and lungs were excised and weighed. The plasma N-terminal pro-brain natriuretic peptide (NT-proBNP) levels were measured with a commercially available enzyme-linked immunosorbent assay (ELISA) kit (SEA489Mu, Cloud-Clone, TX, USA) using a slightly modified, full-length mouse NT-proBNP peptide (custom-made, Scrum, Tokyo, Japan) as the standard. In a separate experiment, CSQ-tg mice were administered either vehicle (n = 18) or TAK-272 (300 mg/kg, n = 18) from 5 weeks of age, and blood samples were collected after 16 or 17 days of drug administration under isoflurane-anesthesia. The hearts were excised after their sacrifice. The plasma cardiac troponin I (cTnI) levels were measured using a commercially available ELISA kit (CTNI-1-US, Life Diagnostics, PA, USA). The left ventricles was placed in RNA*later*^®^ (AM7021, Thermo Fisher Scientific K.K., Japan) solution and stored at 4°C prior to RNA extraction. Echocardiography was conducted with the other set of animals using Vevo 2100 ultrasound system (VisualSonics, ON, Canada). CSQ-tg mice were randomized based on their body weights, left ventricular (LV) end diastolic diameter (EDD) and LV ejection fraction (EF) at 5 weeks of age. Then, they were administered either vehicle (n = 12) or TAK-272 (300 mg/kg, n = 12) from 5 weeks of age. After 12 or 13 days of treatment, their LV short axis M-mode view at papillary muscle level was obtained. LVEF was calculated by Teichholz’s formula. A group of wild-type (WT) mice treated with vehicle was also used in this study. We used three WT mice since the variation of ejection fraction and left ventricular end diastolic diameter of WT mice were quite small as compared with CSQ-tg mice in our preliminary studies.

### Plasma renin activity and blood pressure

The effect of TAK-272 on the Plasma renin activity (PRA) was determined by treating CSQ-tg mice with either vehicle (n = 10) or TAK-272 (300 mg/kg, n = 10) from 5 weeks of age and collecting the blood samples from the tail vein using EDTA 24 h after 7 days of drug administration. The PRA was measured using a commercially available radioimmunoassay kit (Fujirebio, Japan). In a separate experiment, the blood pressure (BP) was determined after treating CSQ-tg mice with either vehicle (n = 8) or TAK-272 (300 mg/kg, n = 8) from 5 weeks of age. The systolic blood pressure (SBP) was measured by the tail-cuff method using a BP-98A blood pressure analyzer (Softron, Tokyo, Japan) under blind conditions, 24 h after 7 days of drug administration. A group of WT mice (n = 5) treated with vehicle were also used in each study.

### Comparison of the efficacy of TAK-272 and aliskiren

The CSQ-tg mice were treated with vehicle (n = 8), TAK-272 (300 mg/kg, n = 8), or aliskiren (300 mg/kg, n = 8) from 5 weeks of age, in order to compare the effects of TAK-272 and aliskiren at the same dose level on the PRA. Blood samples were collected pre-dose and 2 and 24 h after the administration of a single dose, and the PRA was measured as already described. The cardioprotective effects of the two drugs were compared by treating the CSQ-tg mice with vehicle (n = 12), TAK-272 (300 mg/kg, n = 12), or aliskiren (300 mg/kg, n = 12). A group of WT mice (n = 4) were also used in each study. The blood and tissue samples were collected and weighed, and the plasma NT-proBNP levels were measured as already described, after 19 days of drug administration.

### Comparison of the renin inhibitory activity of TAK-272 and aliskiren *in vitro*

To obtain human plasma, blood was collected from in-house volunteers using EDTA with approval by the Research Ethics Committee of Takeda Pharmaceutical Company Limited. Mouse plasma was purchased from Charles River Laboratories Japan, Inc. TAK-272 or aliskiren was dissolved and diluted with DMSO/saline (50 v/v%) to prepare the compound solution (0.5 nmol/l to 500 mmol/l) and then the solution was mixed with plasma in the ratio of 1 : 24. The renin inhibitory activity of TAK-272 and aliskiren on human and mouse plasma was measured as already described.

### Analysis of mRNA expression by real-time polymerase chain reaction (RT-PCR)

Total RNA was extracted using the RNeasy Mini kit (Qiagen, Germany) and converted into cDNA using the High Capacity cDNA Reverse Transcription kit (Life Technologies, USA). The gene expression was analyzed on a 7900HT Fast Real-Time PCR system (Life Technologies, USA) using the TaqMan^®^ Universal Master Mix II (Life Technologies, USA) and primer-probe sets (TaqMan^®^ Gene Expression Assays, Life Technologies, USA) against NADPH oxidase 4 (Nox4, Mm00479246_m1), nitric oxide synthase 3 (Nos3, Mm00435217_m1), transforming growth factor-β1 (TGF-β1, Mm01178820_m1), collagen type I α1 (Col1α1, Mm00801666_g1), and β-actin (ACTB, Mm02619580_g1). ACTB was used as an endogenous control gene. The relative gene expression values were calculated by the ΔΔCT method.

### Statistical analysis

All data except for the *in vitro* renin inhibitory activity of compounds are expressed as the mean ± S.D. All statistical analyses except for the *in vitro* renin inhibitory activity of compounds were performed using the EXSUS software (version 8.0.0, CAC Croit, Tokyo, Japan). The data were firstly analyzed by F-test for homogeneity of variance, then Student’s t-test (*P* > 0.20 by F-test) or Aspin-Welch’s *t*-test (*P* < 0.20 by F-test) was conducted for a comparison between two groups. For a comparison between more than three groups, the data were firstly analyzed by Bartlett’s test for homogeneity of variance, then one-way ANOVA followed by Dunnett’s test or Williams’ test (*P* > 0.05 by Bartlett's test), or Kruskal-Wallis non parametric analysis of variance followed by Steel’s test or Shirley-Williams’ test (*P* < 0.05 by Bartlett's test) was conducted. A survival analysis was performed using the Kaplan–Meier method with log-rank test. If needed, Bonferroni correction was additionally conducted to avoid multiplicity. The detailed statistical methods are described in the figure and table legends. The IC_50_ values and its 95% confidence interval (CI) of compounds for the renin inhibitory activity *in vitro* were calculated by logistic regression analysis using SAS software (SAS Institute Japan Ltd., Tokyo, Japan).

## Results

### Mortality

**Fig 1:**
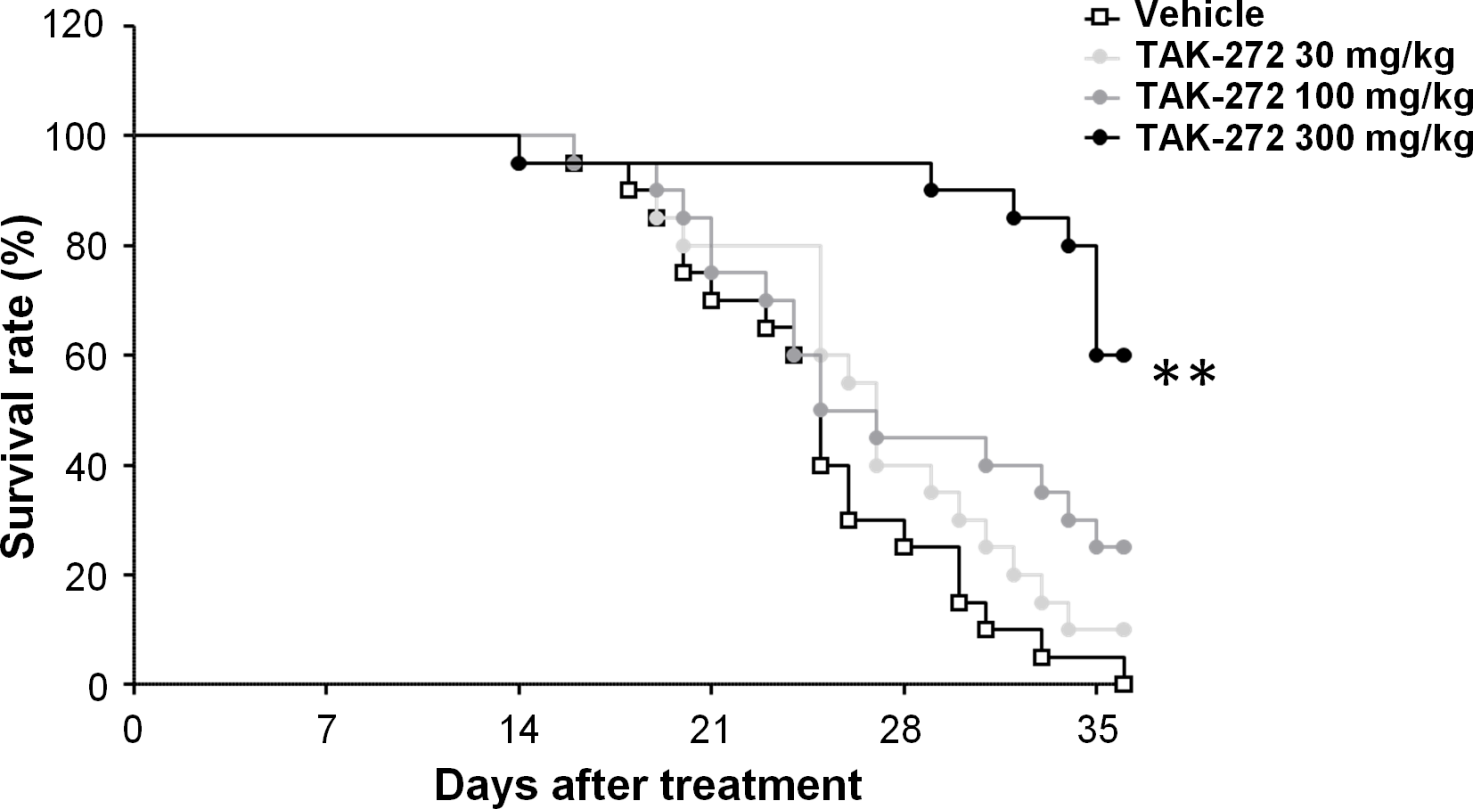
Survival rates of calsequestrin transgenic (CSQ-tg) mice treated with vehicle or TAK-272. Vehicle, TAK-272 30, 100, or 300 mg/kg were orally treated once daily to CSQ-tg mice. Each treatment group was comprised of 20 animals. The number of animals which were euthanized or found dead is the following; Vehicle (E: 16, F: 4), TAK-272 30 mg/kg (E: 17, F: 1), 100 mg/kg (E: 9, F: 6), 300 mg/kg (E: 2, F: 6) (E: euthanasia, F: found dead). **P < 0.01 vs. vehicle by Kaplan–Meier survival analysis with a log-rank test followed by the Bonferroni correction.

The effects of TAK-272 on the survival rates of CSQ-tg mice were evaluated. All vehicle-treated CSQ-tg mice were died or euthanized (due to pre-defined moribund conditions) within 36 days from the start of the treatment. The survival rate of TAK-272-treated mice was significantly (TAK-272 300 mg/kg: *P* < 0.01 vs. vehicle) prolonged in a dose-dependent manner at 36 days from the start of the treatment. (Fig 1).

### Cardiac hypertrophy and plasma heart failure marker level

TAK-272 (300 mg/kg) significantly reduced the left and right ventricular and right atrial weights in CSQ-tg mice (*P* < 0.005 vs. vehicle in each tissue) after 19 days of drug administration (Table 1). TAK-272 (100 and 300 mg/kg) also reduced the lung weight significantly (100 mg/kg: *P* < 0.025, 300 mg/kg: *P* < 0.005 vs. vehicle). These anti-hypertrophic effects of TAK-272 (300 mg/kg) were accompanied by a significant reduction (26%) in the plasma NT-proBNP level (a heart failure biomarker) (*P* < 0.025 vs. vehicle). These results indicated that the improvement of cardiac failure led to the reduction of mortality in CSQ-tg mice.

**Table 1:** Effects of TAK-272 on cardiac hypertrophy and plasma heart failure biomarker level.

Parameter	Group (n)			
	Vehicle	TAK-272	TAK-272	TAK-272
		30 mg/kg	100 mg/kg	300 mg/kg
	(7–8)	(9)	(10)	(9)
**Body weight (g)**	15.1 ± 2.2	16.8 ± 2.8	15.1 ± 1.8	16.3 ± 3.2
**Left ventricle (mg)**	150.2 ± 13.9	145.2 ± 8.8	142.4 ± 9.2	132.3 ± 9.3**
**Right ventricle (mg)**	44.2 ± 3.4	41.6 ± 5.7	41.6 ± 2.9	37.8 ± 5.6**
**Left atrium (mg)**	10.8 ± 1.1	10.4 ± 5.1	10.4 ± 1.2	8.5 ± 2.6
**Right atrium (mg)**	8.1 ± 1.0	7.3 ± 2.2	7.1 ± 1.2	5.8 ± 1.8**
**Lung (mg)**	137.8 ± 12.9	128.5 ± 14.1	126.2 ± 8.3*	113.1 ± 10.9**
**NT-proBNP (ng/ml)**	9.9 ± 1.8	7.9 ± 2.8	9.2 ± 2.0	7.4 ± 2.4^a^

The body weights, cardiac tissue and lung weights, and plasma N-terminal pro-brain natriuretic peptide (NT-proBNP) levels of calsequestrin transgenic (CSQ-tg) mice orally treated with either vehicle or TAK-272 (30, 100, and 300 mg/kg) are indicated. Data are expressed as the mean ± S.D. The number of deaths in each group during the study period was as follows: 2, 1, 0, and 1 in vehicle (n = 7–8), 30 mg/kg (n = 9), 100 mg/kg (n = 10), and 300 mg/kg (n = 9) of TAK-272 groups, respectively. The plasma sample of one animal in the vehicle-treated group could not be obtained due to a technical problem. Therefore, the body/tissue weight and plasma NT-proBNP level were measured in 8 and 7 animals in this group, respectively.

*P < 0.025

**P < 0.005 vs. vehicle by one-tailed Williams' test.

### Echocardiography

Echocardiography was conducted to assess the left ventricular contractility and dilation. Before evaluating the effects of TAK-272, we conducted a preliminary study with the same time course as present study comparing WT and CSQ-tg mice twice. As being well consistent with these studies, we confirmed in the present study that LVEDD of CSQ-tg mice was already larger than that of WT mice, while LVEF of CSQ-tg mice was comparable to that of WT mice at 5 weeks of age. Then, LVEF of CSQ-tg mice was severely decreased in two weeks. TAK-272 (300 mg/kg) significantly inhibited the decrease of LVEF in CSQ-tg mice (*P* < 0.01 vs. vehicle) after 2 weeks of drug administration (Table 2). This result suggested that the reduction of mortality in CSQ-tg mice was accompanied with the improvement of systolic function.

**Table 2:** Effects of TAK-272 on echocardiographic parameters.

Parameter	Age (weeks)	Group (n)		
		WT	CSQ-tg	CSQ-tg
		Vehicle	Vehicle	TAK-272
		(3)	(10)	(12)
**LVEF (%)**	**5**	74.1 ± 5.6	64.2 ± 9.5	64.5 ± 9.6
	**7**	82.7 ± 2.4	13.0 ± 7.1^##^	26.7 ± 9.8*
**LVEDD (mm)**	**5**	3.3 ± 0.4	4.3 ± 0.3^#^	4.3 ± 0.3
	**7**	3.3 ± 0.4	5.3 ± 0.5^##^	5.0 ± 0.4

The left ventricular (LV) ejection fraction (EF) and LV end diastolic diameter (EDD) of wild-type (WT) (n = 3) and calsequestrin transgenic (CSQ-tg) mice orally treated with vehicle (n = 10) or TAK-272 (300 mg/kg, n = 12) are indicated. Data are expressed as the mean ± S.D. two mice in vehicle group were dead during the study period.

^#^*P* < 0.05

^##^*P* < 0.01 vs. WT at same age by Aspin-Welch's *t*-test.

*P < 0.01 vs. CSQ-tg + vehicle at 7 weeks of age by Aspin-Welch's *t*-test.

### Plasma renin activity and blood pressure

**Fig 2:**
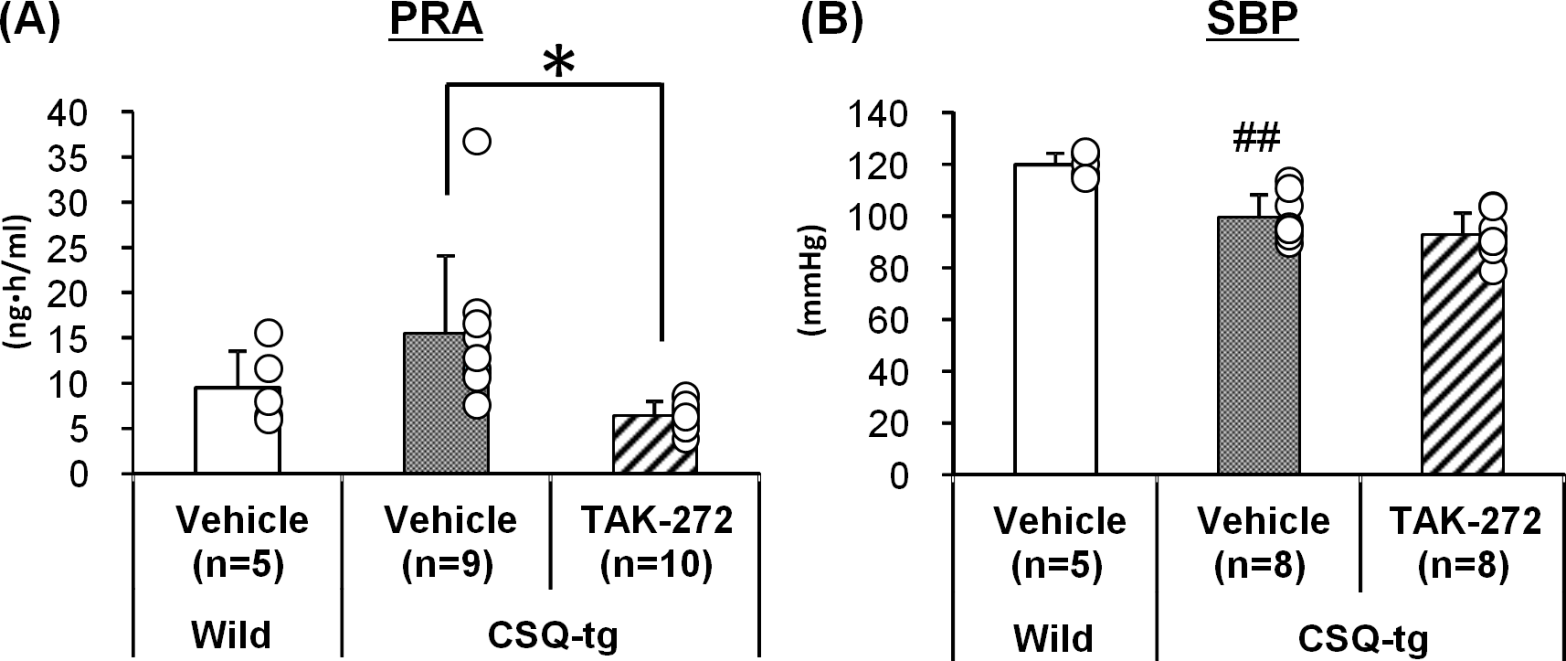
Effects of TAK-272 on the plasma renin activity (PRA) and blood pressure (BP). The PRA (A) and systolic BP (SBP) (B) of wild-type (WT) and calsequestrin transgenic (CSQ-tg) mice at 24 h after 7 days of treatment with vehicle or TAK-272 (300 mg/kg) are indicated. The number of deaths in each group during the PRA study was as follows: 0, 1, and 0 in WT, CSQ-tg + vehicle, and 300 mg/kg TAK-272 groups, respectively. No death was observed in the BP study groups. Data are expressed as the mean + S.D. ^##^*P* < 0.01 vs. WT by Student's *t*-test, *P < 0.05 vs. CSQ-tg + vehicle by Aspin-Welch's *t*-test.

TAK-272 (300 mg/kg) significantly reduced the PRA 24 h after the 7-day treatment in CSQ-tg mice (Fig 2A). The mean PRA of mice in the WT, vehicle, and TAK-272 groups was 9.5 ± 4.1, 15.5 ± 8.6, and 6.4 ± 1.6 ng·ml/h, respectively. Further, BP of mice was measured under conscious conditions in order to determine the contribution of afterload reduction to the TAK-272-mediated improvement in survival rate (Fig 2B). TAK-272 (300 mg/kg) did not reduce the SBP 24 h after the 7-day treatment in CSQ-tg mice. The mean SBP of mice in the WT, vehicle, and TAK-272 groups was 120 ± 4, 99 ± 9, and 93 ± 8 mmHg, respectively.

### RAS-related genes expression and a cardiac injury marker

**Fig 3:**
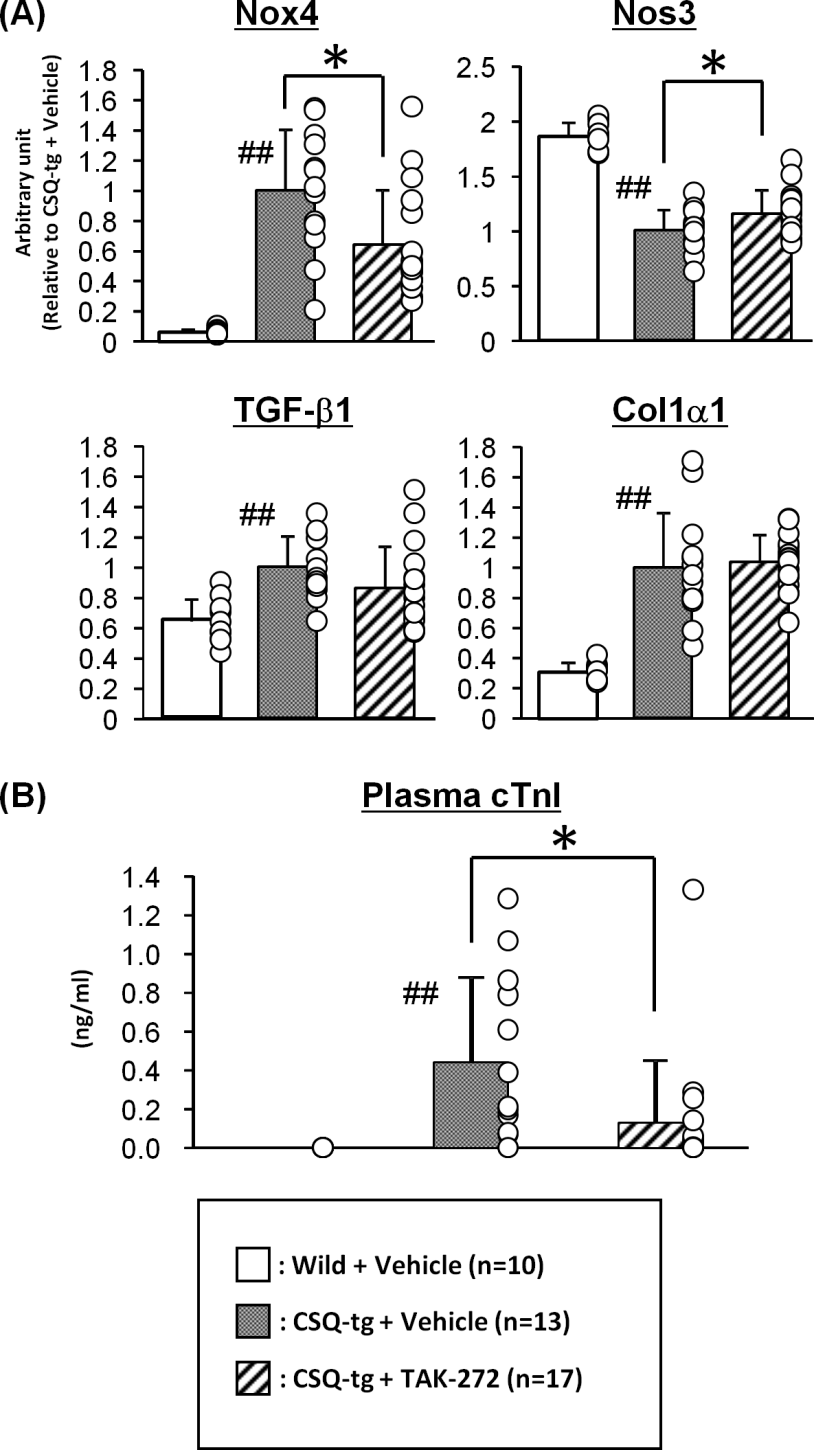
Effects of TAK-272 on the expression of renin angiotensin system (RAS)-related genes and cardiac injury markers. The left ventricular NADPH oxidase 4 (Nox4), nitric oxide synthase 3 (Nos3), transforming growth factor-β1 (TGF-β1), and collagen type I α1 (Col1α1) expression (A) and plasma cardiac troponin I (cTnI) levels (B) in wild-type (WT) and calsequestrin transgenic (CSQ-tg) mice after 16–17 days of treatment with vehicle or TAK-272 (300 mg/kg) are indicated. The number of deaths in each group during the study period was as follows: 0, 5, and 1 in WT, CSQ-tg + vehicle, and 300 mg/kg TAK-272 groups, respectively. Data are expressed as the mean + S.D. ^##^*P* < 0.01 vs. WT by Aspin-Welch's *t*-test or Student's *t*-test, *P < 0.05 vs. CSQ-tg + vehicle by Student's *t*-test.

The effects of TAK-272 on RAS-related gene expression and cardiomyocyte injury were investigated. The expression of cardiac Nox4, TGF-β1, and Col1α1 mRNA was significantly higher, and the expression of cardiac Nos3 mRNA was significantly lower in CSQ-tg mice than in WT mice (*P* < 0.01 in each gene) (Fig 3A). Further, the expression of the fibrosis markers, TGF-β1 and Col1α1 mRNA, was not altered by TAK-272 (300 mg/kg) treatment. However, TAK-272 significantly attenuated the increase of the expression of the oxidative stress marker, Nox4 (*P* < 0.05 vs. vehicle-treated CSQ-tg mice), and the endotherial NO synthase, Nos3 (*P* < 0.05 vs. vehicle-treated CSQ-tg mice), mRNA. Additionally, the plasma levels of the cardiac injury marker, cTnI, which were dramatically elevated in CSQ-tg mice as compared to WT mice (*P* < 0.01), were also significantly reduced by TAK-272 (*P* < 0.05 vs. vehicle-CSQ-tg mice) (Fig 3B).

### Comparison of the cardioprotective efficacy of TAK-272 and aliskiren

**Fig 4:**
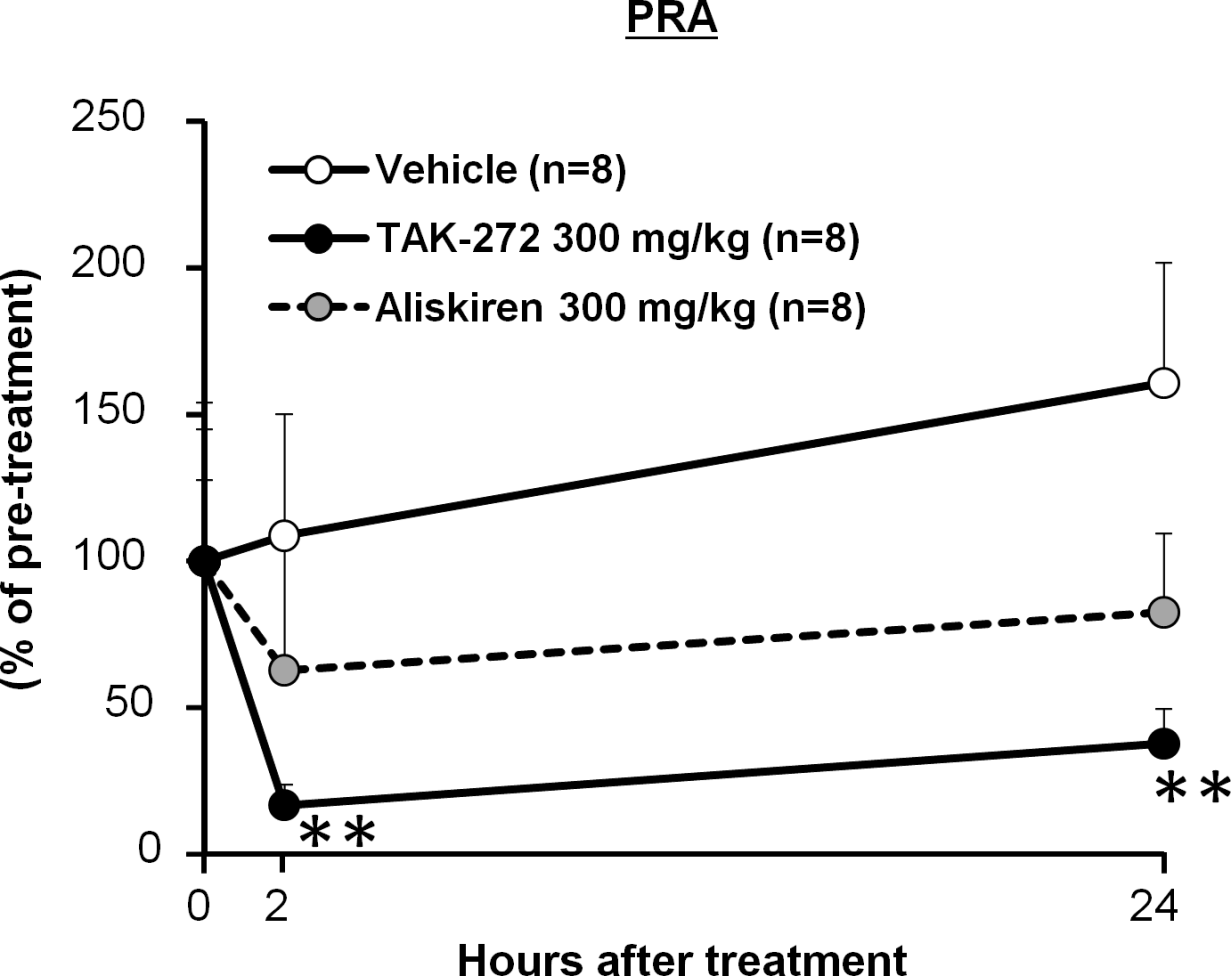
Comparison of the efficacy of TAK-272 and aliskiren with respect to plasma renin activity (PRA) inhibition. The PRA in calsequestrin transgenic (CSQ-tg) mice after single oral administration of vehicle, TAK-272 (300 mg/kg), or aliskiren (300 mg/kg) is indicated. Data are expressed as the mean + S.D. **P < 0.01 vs. vehicle by Dunnett's test followed by the Bonferroni correction.

The cardioprotective efficacy of the two DRIs, TAK-272 and aliskiren, was compared by evaluating their effects on cardiac hypertrophy and the plasma NT-proBNP levels in CSQ-tg mice, after 19 days of treatment with the compounds (300 mg/kg). TAK-272 significantly and sustainably inhibited the PRA over 24 h after the administration of a single dose (2 h and 24 h: *P* < 0.01 vs. vehicle) (Fig 4) and reduced the heart and lung weights (Right ventricular and lung weights: *P* < 0.05, left and right atrial weights: *P* < 0.01 vs. vehicle) (Table 3) after 19 days of treatment. TAK-272 also significantly decreased the plasma NT-proBNP level. In this study, we did not observe the reduction in LV weight, probably because deaths of three mice in vehicle group during treatment period caused the less detectability. Although the renin inhibitory activity of aliskiren for mouse renin was more potent than that of TAK-272 *in vitro* (Table 4), aliskiren slightly inhibited the PRA at 2h and 24h which was less potent than that observed with TAK-272 (Fig 4). Moreover, the tissue weights and plasma NT-proBNP level were not reduced by aliskiren (Table 3). The IC_50_ values for the renin inhibitory acivity of TAK-272 and aliskiren in vitro on the human plasma renin was potent, and the IC_50_ value was 2.1 nmol/L, similar to that of aliskiren (0.8 nmol/L) (Table 4). The inhibitory effect of TAK-272 on mouse plasma renin was less potent than those of aliskiren.

**Table 3:** Comparison of the cardioprotective efficacy of TAK-272 and aliskiren with respect to cardiac hypertrophy and plasma heart failure biomarker level.

Parameter	Group (n)			
	WT	CSQ-tg		
	Vehicle	Vehicle	TAK-272	Aliskiren
			300 mg/kg	300 mg/kg
	(4)	(8)	(11)	(12)
**Body weight (g)**	19.6 ± 0.6	15.8 ± 1.5^##^	19.1 ± 1.0**	16.6 ± 2.3
**Left ventricle (mg)**	67.4 ± 1.4	154.4 ± 15.8^##^	150.0 ± 9.2	150.2 ± 11.6
**Right ventricle (mg)**	14.5 ± 1.4	46.0 ± 6.8^##^	40.6 ± 5.3*	45.5 ± 3.0
**Left atrium (mg)**	2.1 ± 0.3	14.3 ± 3.8^##^	8.2 ± 3.0**	11.4 ± 2.2
**Right atrium (mg)**	2.9 ± 0.8	8.1 ± 1.8^##^	5.3 ± 0.8*	7.9 ± 2.2
**Lung (mg)**	120.5 ± 7.4	138.7 ± 15.0^#^	121.3 ± 10.1*	129.9 ± 13.6
**NT-proBNP (ng/ml)**	1.0 ± 0.1	8.5 ± 3.5^##^	4.5 ± 1.3*	7.7 ± 2.3

The body weights, cardiac tissue and lung weights, and plasma N-terminal pro-brain natriuretic peptide (NT-proBNP) levels of wild-type (WT) (n = 4) and calsequestrin transgenic (CSQ-tg) mice orally treated with vehicle (n = 8), TAK-272 (300 mg/kg, n = 11), or aliskiren (300 mg/kg, n = 12) are indicated. Data are expressed as the mean ± S.D. The number of deaths in each group during the study period was as follows: 0, 4, 1, and 0 in WT, vehicle, TAK-272, and aliskiren groups, respectively.

^#^*P* < 0.05

^##^*P* < 0.01 vs. WT, by Student's *t*-test or Aspin-Welch's *t*-test

*P < 0.05

**P < 0.01 vs. CSQ-tg + vehicle by Dunnett's test or Steel's test.

**Table 4:** Species difference of TAK-272 and aliskiren for IC_50_ values of plasma renin activity inhibition.

	IC_50,_ nmol/L (95% CI)	
	Human	Mouse
**TAK-272**	2.1 (1.7–2.6)	53 (40–69)
**Aliskiren**	0.8 (0.7–1.0)	9 (6–12)

The IC_50_ values and its 95% confidence interval (CI) of TAK-272 and aliskiren for the renin inhibitory activity *in vitro* are indicated. IC_50_ values are calculated from dose response curves using 2 replicates for each of 6 concentrations of TAK 272 or aliskiren.

## Discussion

CSQ-tg mice have been reported to show severe cardiac dysfunction and hypertrophy and undergo premature death [23–25] which was consistent with this report. In addition, RAS inhibitors showed their cardioprotective effect accompanied with reduced mortality in CSQ tg mice [25, 26]. Our experiment demonstrated significant increases in angiotensin II level in both plasma and cardiac tissue (S1 Fig), and also a slight elevation of PRA (Fig 2), indicating RAS activation in CSQ-tg mice similar to heart failure patients. From these findings, CSQ-tg mice are considered as a physiologically relevant model to investigate the effect of RAS inhibitors including DRI on heart failure.

Several mechanisms have been proposed to date to account for the beneficial effects of RAS inhibitors in heart failure, with afterload reduction being considered one of the contributors to their cardioprotective effects [27]. Although TAK-272 (300 mg/kg) persistently inhibited the PRA in CSQ-tg mice, a significant reduction in the SBP was not observed 24 h after repetitive drug administration. That is probably due to the lower blood pressure levels of CSQ tg mice than those of normal control mice. Consistently, it was reported that RAS inhibitors showed minimal blood pressure lowering effect in normotensive subjects [28, 29]. Our result was also consistent with the previous reports that aliskiren showed cardioprotective effects without lowering blood pressure in normotensive heart failure mice, despite showing remarkable increase in cardiac renin concentration due to renin inhibition [19]. These results imply that mechanisms other than afterload reduction may be contributing to the cardioprotective effects of TAK-272. We therefore investigated the effects of TAK-272 on RAS-related genes expression to determine the underlying beneficial mechanism. TGF-β signaling, which is widely known to act downstream of angiotensin II, plays a crucial role in cardiac remodeling and fibrosis [30]. However, the expression of TGF-β and Col1α1 mRNA was not altered by TAK-272 in our study. CSQ-tg mice develop cardiac dysfunction which could induce not only the activation of RAS signaling with renin secretion stimulated by decreased renal perfusion, but also the dysfunction of β-adrenergic signaling [23]. We speculate that TGF-β and collagen might be up-regulated in CSQ-tg mice by not only increased angiotensin II signaling, but also other mechanisms including uncoupled β-adrenergic signaling followed by elevated β-arrestin expression in CSQ-tg mice [31, 32], so that TAK-272 failed to decrease TGF-β and collagen 1A1 mRNA. On the other hand, increased oxidative stress is also well known to contribute to the pathological processes of heart failure, such as apoptosis, fibrosis, and remodeling [33]. NADPH oxidases (Noxs) are considered one of the major sources of reactive oxygen species. Nox2 and Nox4 are predominantly expressed in the heart [33]. The Nox4 mRNA expression was markedly elevated while the Nox2 mRNA expression was unaltered in the cardiac tissues of CSQ-tg mice, as compared with that of WT mice (data not shown). A previous report showed that Nox4 expression in cardiomyocytes is upregulated in response to cardiac stresses [34], In the present study, TAK-272 significantly decreased the Nox4 mRNA expression in the cardiac tissues of CSQ-tg mice, probably causing a reduction in reactive oxygen species production. The cardiac Nox4 expression and Nox activity have been reported to be elevated by angiotensin II [34, 35]. Hence, the suppression of RAS signaling by renin inhibitors is a plausible explanation for the observed decrease in Nox4 expression. Further, the expression of endothelial nitric oxide synthase, Nos3, have been showed to be reduced by angiotensin II, and restored by RAS inhibitors [36, 37]. NO generated by Nos3 plays an important role to maintain vascular homeostasis, and it was reported that the overexpression of Nos3 improved the survival and lung congestion of a heart failure mice model [38]. It is speculated that the enhanced NO production with the increase in Nos3 expression by TAK-272 treatment might act as an oxidant scavenger for cardioprotective effect as shown in that report. In our study, the plasma cTnI levels were significantly decreased in TAK-272-treated animals, indicating a reduction in cardiomyocyte death. Thus, the inhibition of reactive oxygen species production and the improvement of coronary vascular homeostasis via the restoration of cardiac Nox4 and Nos3 expression might contribute to the cardioprotective effects of TAK-272. Our experiment indicated the reduction of angiotensin II level after two weeks treatment of TAK-272 not only in plasma but also in cardiac tissue (S1 Fig). The local cardiac AII reduction by TAK-272 could at least partially explain the changes of gene expressions.

When the cardioprotective efficacy of TAK-272 and aliskiren was compared by oral gavage administration, TAK-272 showed beneficial effects against cardiac hypertrophy and reduced the heart failure biomarker level, whereas aliskiren failed to show similar efficacy at the same dose as TAK-272. It is possible that the fast progression of cardiac dysfunction in CSQ-tg mice as shown by echocardiography is one of reasons why orally administered aliskiren failed to show the efficacy despite using higher dose in our study as compared to reported studies [17–19]. The difference in the efficacy of the two DRIs in our experimental condition is primarily explained by the difference in their renin inhibitory potential *in* vivo after oral gavage. In the present study, the PRA in CSQ-tg mice was inhibited more potently and persistently by TAK-272 than by aliskiren, which has very low oral bioavailability (BA) in rodents, at the same dose. It is reported that oral BA of aliskiren is also very low in humans [39]. There were many clinical trials of aliskiren in heart failure patients such as ASTRONAUT, ALOFT, ASPIRE, ATMOSPHERE. In these clinical trials, increase in incidence of adverse effects such as hypotension, hyperkalemia, and kidney failure that were found in patients treated with aliskiren plus another RAS inhibitor [40–43]. Since it is considered that these AEs were caused by an excess dual RAS blockade with high doses of its inhibitors including renin inhibitor, they should be used as monotherapy in heart failure patients with systolic dysfunction. In this case, because of the low oral BA, aliskiren will show insufficient renin inhibition to exert the maximum efficacy of a renin inhibitor as monotherapy. On the other hand, TAK-272 has desirable oral PK profiles so that TAK-272 might inhibit PRA more potently and persistently than aliskiren in clinical as similar as in our study [16]. In the ATMOSPHERE study, which was the largest clinical trial to evaluate the cardioprotective effect of aliskiren in heart failure with reduced ejection fraction patients, aliskiren failed to meet the prespecified criteria for noninferiority to an evidence-based dose of enalapril, an ACE inhibitor [42]. One possible reason for this unfortunate result is insufficient renin inhibition by the moderate doses of aliskiren used in this study (mean doses: 273–278 mg/day). A previous report indicated that the BP lowering effect and compensated elevation of plasma renin concentration observed with 600 mg/day of aliskiren were more potent than those observed with 300 mg/day of aliskiren, suggesting that higher doses of aliskiren than those used in the ATMOSPHERE study could cause more robust renin inhibition [44]. However, the clinical dose of aliskiren is limited to less than 300 mg/day, owing to a significantly increased incidence of diarrhea at 600 mg/day. As TAK-272 has favorable oral BA, it is expected to inhibit renin more potently at lower doses than aliskiren with fewer possibilities of diarrhea due to high dose, and exert the maximum efficacy of a renin inhibitor beyond the efficacy of aliskiren. Renin controls the first and rate-limiting step in the RAS pathway, and the inhibition of this enzyme should efficiently block the RAS cascade [13, 14]. Further, it is expected that TAK-272 could demonstrate robust and sustained PRA inhibition as an oral drug via its favorable oral PK profile and potent renin inhibitory activity not only in mice but also in human beings. Thus, it is possible that TAK-272 could show more potent cardioprotective effect than other RAS inhibitors, an ACE inhibitor or an ARB, in clinical study, and TAK-272 become a first-line RAS inhibitor for the treatment of heart failure. In the present study, we demonstrated that aliskiren showed a more potent *in vitro* renin inhibitory activity than TAK-272. Therefore, aliskiren possibly shows cardioprotective effects if it is administered by other routes than oral. Indeed, the potent cardioprotective effects of aliskiren have been proven in several animal models by non-oral administration routes such as intraperitoneal dosing [45]. Zhi *et al*. also reported that aliskiren, which is administered by subcutaneously implanted osmotic minipump, inhibited homocysteine-induced myocardial fibrosis in mice [46].

One of limitations of this study is that we used a high dose of renin inhibitors. The renin inhibitory activity of TAK-272 and aliskiren in mice is more than ten times weaker than that in human beings due to species-specific differences on the amino acid sequence of the catalytic domain of renin [47, 48]. We cannot exclude the possibility that off-target effects related to high doses might affect the results of the present study. As another limitation, while we found a tendency of dose-dependent PRA inhibition with aliskiren from 30 to 300 mg/kg in our study (S2 Fig), we did not evaluate PRA inhibition using over 300 mg/kg of aliskiren by oral administration due to safety concerns with high dose. Therefore, we also cannot exclude the possibility that that we were not able to measure maximum effect of aliskiren in our setting. Thus, clinically relevant models such as monkey or human renin and angiotensinogen transgenic mice should be used to conduct further pharmacological studies.

In conclusion, TAK-272 significantly reduced the mortality of the CSQ-tg heart failure mouse model. This efficacy was induced by the improvement of cardiac failure accompanied with protection against cardiac hypertrophy, prevention from systolic dysfunction, and reduced the plasma NT-proBNP level. The therapeutic effects of TAK-272 may be mediated by the inhibition of reactive oxygen species production and the improvement of coronary vascular homeostasis via the restoration of cardiac Nox4 and Nos3 expression. Further, TAK-272 inhibits the PRA, whose elevation contributes to the progression of heart failure, potently and persistently. Thus, our findings indicate that TAK-272 treatment can be a new attractive therapy that could improve the survival and symptoms of heart failure patients via robust RAS inhibition.

## Supporting information

S1 Fig Effects of TAK-272 on plasma and cardiac angiotensin II level. Plasma and cardiac angiotensin II (AII) level of wild-type (WT) (n = 8) and CSQ-tg mice after two weeks treatment with vehicle (n = 8) or TAK-272 (300 mg/kg, n = 7) are indicated. The number of deaths in each group during the study period was as follows: 0, 0, and 1 in WT, CSQ-tg + vehicle, and 300 mg/kg of TAK-272 group, respectively. After two weeks of drug administration, blood samples were collected from the abdominal vein using EDTA (15575020, Thermo Fisher Scientific K.K., Japan) (at a final concentration of 3 mM) as an anticoagulant under anesthesia with 2–3% isoflurane (Mylan, UK). The hearts were excised after sacrificing the animals with bleeding. Plasma and cardiac AII level were measured by a commercial EIA kit (Bertin Pharma, France). Data are expressed as the mean + S.D. ^#^*P* < 0.05, ^##^*P* < 0.01 vs. WT by Aspin-Welch's *t*-test or Student's *t*-test, **P < 0.01 vs. CSQ-tg + vehicle by Aspin-Welch's *t*-test or Student's *t*-test,.
(PPTX)

S2 Fig Dose-dependent PRA inhibition by aliskiren. The PRA of calsequestrin transgenic (CSQ-tg) mice at 2 and 24 h after single treatment with vehicle, aliskiren (30, 100, and 300 mg/kg) or TAK-272 (300 mg/kg) are indicated (n = 8 in each group). Drugs were administered by oral gavage at 5 weeks of age and collecting the blood samples from the tail vein using EDTA 2 and 24 h after single drug administration. The PRA was measured using a commercially available radioimmunoassay kit (Fujirebio, Japan). Data are expressed as the mean + S.D. **P < 0.01 vs. vehicle by Dunnett's test followed by the Bonferroni correction.
(PPTX)
