# Supplementary material for: TAK-272 (imarikiren), a novel renin inhibitor, improves cardiac remodeling and mortality in a murine heart failure model
Source: PLoS One. 2018 Aug 9;13(8):e0202176. doi: 10.1371/journal.pone.0202176 (PMC6084973; doi:10.1371/journal.pone.0202176)
Supplement: S1 Fig — Plasma and cardiac angiotensin II (AII) level of wild-type (WT) (n = 8) and CSQ-tg mice after two weeks treatment with vehicle (n = 8) or TAK-272 (300 mg/kg, n = 7) are indicated. The number of deaths in each group during the study period was as follows: 0, 0, and 1 in WT, CSQ-tg + vehicle, and 300 mg/kg of TAK-272 group, respectively. After two weeks of drug administration, blood samples were collected from the abdominal vein using EDTA (15575020, Thermo Fisher Scientific K.K., Japan) (at a final concentration of 3 mM) as an anticoagulant under anesthesia with 2–3% isoflurane (Mylan, UK). The hearts were excised after sacrificing the animals with bleeding. Plasma and cardiac AII level were measured by a commercial EIA kit (Bertin Pharma, France). Data are expressed as the mean + S.D. #P < 0.05, ##P < 0.01 vs. WT by Aspin-Welch's t-test or Student's t-test, **P < 0.01 vs. CSQ-tg + vehicle by Aspin-Welch's t-test or Student's t-test,. (PPTX) [file pone.0202176.s001.pptx]

## Slide 1
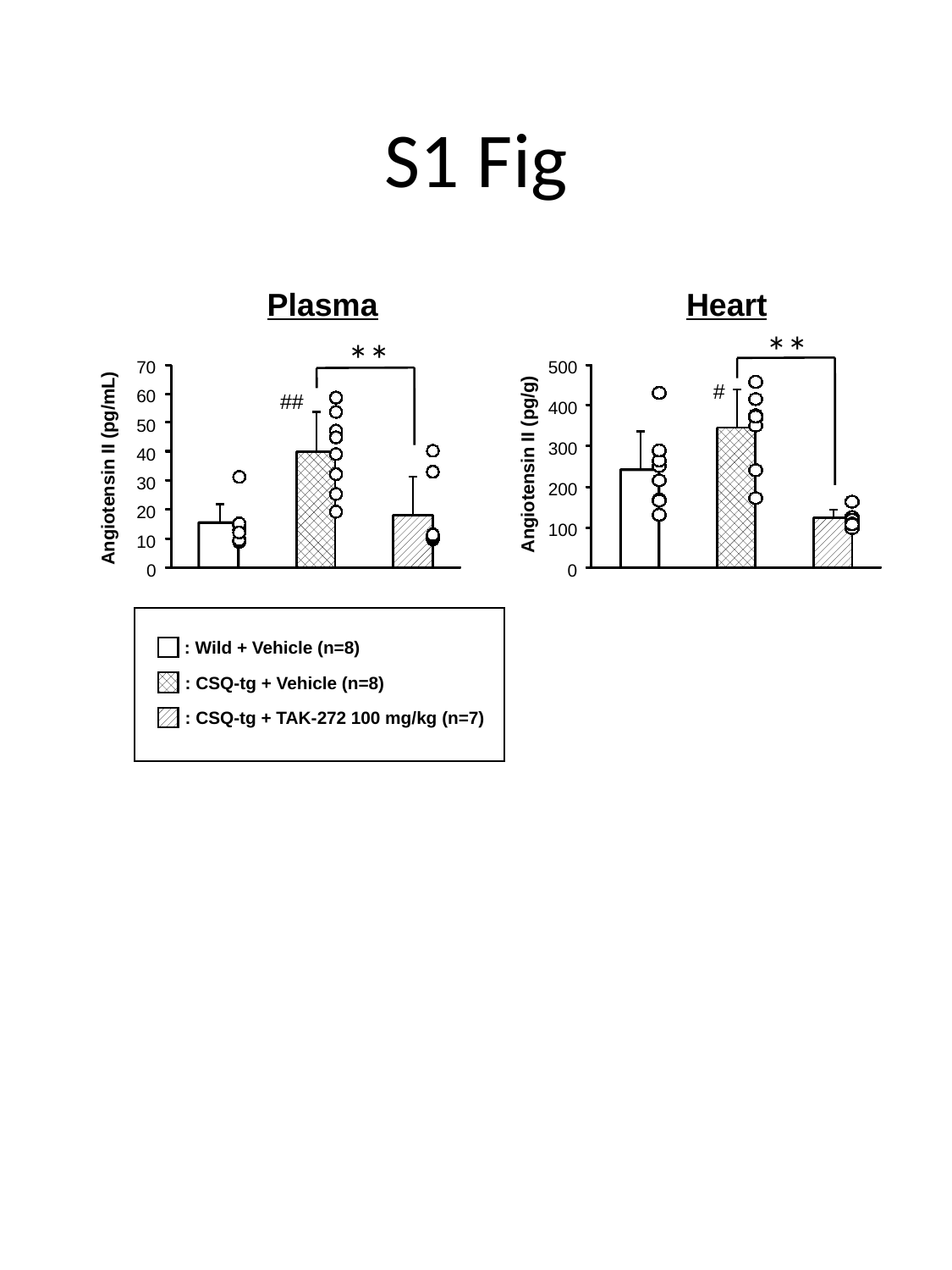

# S1 Fig
Plasma
Heart
**
**
70
500
#
60
##
400
50
Angiotensin II (pg/g)
Angiotensin II (pg/mL)
300
40
30
200
20
100
10
0
0
: Wild + Vehicle (n=8)
: CSQ-tg + Vehicle (n=8)
: CSQ-tg + TAK-272 100 mg/kg (n=7)
