# Supplementary material for: TAK-272 (imarikiren), a novel renin inhibitor, improves cardiac remodeling and mortality in a murine heart failure model
Source: PLoS One. 2018 Aug 9;13(8):e0202176. doi: 10.1371/journal.pone.0202176 (PMC6084973; doi:10.1371/journal.pone.0202176)
Supplement: S2 Fig — The PRA of calsequestrin transgenic (CSQ-tg) mice at 2 and 24 h after single treatment with vehicle, aliskiren (30, 100, and 300 mg/kg) or TAK-272 (300 mg/kg) are indicated (n = 8 in each group). Drugs were administered by oral gavage at 5 weeks of age and collecting the blood samples from the tail vein using EDTA 2 and 24 h after single drug administration. The PRA was measured using a commercially available radioimmunoassay kit (Fujirebio, Japan). Data are expressed as the mean + S.D. **P < 0.01 vs. vehicle by Dunnett's test followed by the Bonferroni correction. (PPTX) [file pone.0202176.s002.pptx]

## Slide 1
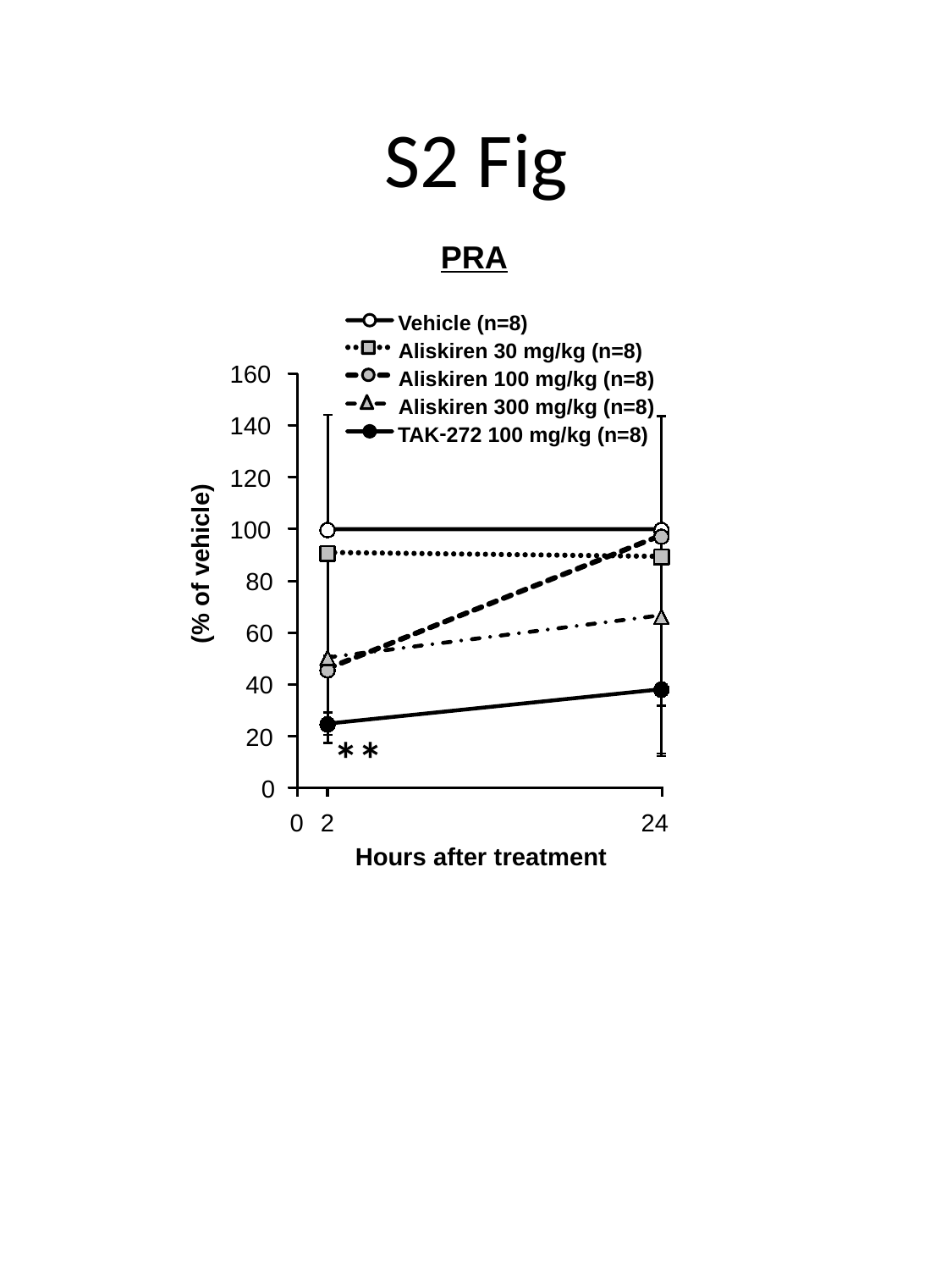

# S2 Fig
PRA
Vehicle (n=8)
Aliskiren 30 mg/kg (n=8)
160
Aliskiren 100 mg/kg (n=8)
Aliskiren 300 mg/kg (n=8)
140
-
TAK
272 100 mg/kg (n=8)
120
(% of vehicle)
100
80
60
40
20
0
0
2
24
Hours after treatment
**
